# Association between Infant Feeding and Early Postpartum Infant Body Composition: A Pilot Prospective Study

**DOI:** 10.1155/2009/648091

**Published:** 2009-03-12

**Authors:** Alex Kojo Anderson

**Affiliations:** Department of Foods and Nutrition, The University of Georgia, 280 Dawson Hall, Athens, GA 30602, USA

## Abstract

Research studies have produced conflicting results of the impact of breastfeeding on overweight/obesity. This study evaluated the impact of infant feeding on infant body composition. There were two groups of mother-infant pairs (exclusive breastfeeding [EBF; *n* = 27] and mixed feeding [MF; *n* = 13]) in this study. At baseline, participants were similar in their demographic characteristics except prepregnancy weight, where MF mothers tended to be heavier than their EBF counterparts (67.3 kg versus 59.9 kg; *P* = .034). Infant birth weight was slightly higher among the MF group than their EBF counterparts (3.5 kg versus 3.4 kg), although the differences were not statistically significant. At 3 months postpartum, mean infant FMI (4.1 kg/m^2^ versus 3.8 kg/m^2^) and percent body fat (24.4% versus 23.1%) were slightly higher among EBF infants than MF infants. In terms of growth velocity, EBF infants gained weight faster than their MF counterparts, although the differences were not statistically significant. The findings from this study suggest that EBF may promote faster weight gain and increase in both fat mass index (FMI) and percent body fat in the early postpartum period in addition to the numerous health benefits enjoyed by the infant and the mother who exclusively breastfeeds her newborn.

## 1. Introduction

A number of primary and review articles provide strong evidence of the protective effect of breastfeeding against overweight/obesity [1–9]. Despite this strong evidence, other studies have reported an inverse relationship or no relationship at all between breastfeeding and overweight/obesity [10]. A 2003 narrative review of the literature by Dewey reported a protective effect of breastfeeding against childhood obesity [11]. Another systematic review by Owen et al. [5] reported an inverse association between breastfeeding and obesity even after adjusting for known confounding factors (parental obesity, maternal smoking, and social class). Other researchers have also observed an inverse association as well as dose-response association between duration of breastfeeding and risk of overweight/obesity [4]. Balaban and Silva in their review have suggested a small, but significant, protective effect of breastfeeding against childhood overweight and obesity [12]. Studies by Wadsworth et al. [13], O'Callaghan et al. [14], and Zive et al. [15] have reported no association between breastfeeding and childhood adiposity, while Agras et al. [16] report breastfeeding as a risk factor for greater adiposity during childhood. While meta-analysis and systematic reviews suggest a small to moderate protection against overweight by breastfeeding, these benefits may not extend to adulthood [3, 4, 11, 12, 17]. Furthermore, a recent randomized control trial conducted in Belarus did not show any benefit of prolonging exclusive breastfeeding on height, BMI, and adiposity during childhood [18]. The inconclusiveness of the literature, therefore, raises the question of how these studies were conducted, indicators of overweight/obesity measured, definition of breastfeeding used, and how infant feeding data were obtained as well as the design of the respective studies. Definition of breastfeeding has not been consistent across the studies, and breastfeeding data have been collected retrospectively, sometimes more than 5 years after the actual practice. Again, overweight/obesity was assessed via body mass index (BMI) and sometimes via skin-fold measurements. Although, the BMI is an appropriate screening tool for overweight/obesity, it is not adequate enough in predicting adiposity which is directly associated with poor health outcomes such as cardiovascular diseases (CVDs). Skin-fold measurements also used to assess adiposity come with a number of associated errors such as technician errors in measurement and instrument error. As such, it is important to consider all these factors in making a conclusive determination regarding the protective effect of breastfeeding against overweight/obesity. Therefore, these limitations of earlier studies evaluating the association between infant feeding and obesity will continue to cast doubts on the relationship between breastfeeding and overweight/obesity.

The purpose of this study was to prospectively examine the association between infant feeding and overweight and adiposity among infants in the early postpartum weeks in a pilot study. This provides a unique opportunity to explore the association between exclusive breastfeeding and infant adiposity measured by the PEA POD body composition system. In addition, unlike earlier studies that assessed breastfeeding data retrospectively, this study assessed infant feeding prospectively alongside body composition measurements.

## 2. Materials and Methods

### 2.1. Study Design

This was a prospective pilot study conducted in Athens-Clarke County and surrounding counties in the state of Georgia between November 2005 and July 2008. The study protocol was approved by the Human Subjects Institutional Review Boards (IRBs) of the University of Georgia (UGA) and the Athens Regional Medical Center (ARMC). Additionally, written informed consent was obtained from mothers before the birth of each participating infant. Subjects were newborns of singleton deliveries of women recruited in their third trimester of pregnancy. Characteristics as well as exclusion and inclusion criteria of mothers are reported elsewhere [19]. Briefly, mothers of these infants were recruited during pregnancy but not later than 36 weeks gestation. Newborns were screened after delivery for inclusion in the present study. Exclusion criteria included preterm (<37 weeks gestation), low birthweight (<2500 grams), and neonates with anomalies requiring them to spend more than 24 hours in the neonatal intensive care unit (NICU).

### 2.2. Study Implementation

Baseline data was collected at the time of recruitment and follow-up at 2, 4, 8, and 12 weeks postpartum. Infant anthropometric and body composition measurements were completed at 2, 4, 8, and 12 weeks postpartum in the Maternal and Child Nutrition Research Lab (MCNRL), Department of Foods and Nutrition, the University of Georgia (UGA), Ga, USA. Mothers of participating infants were interviewed using a structured questionnaire containing both closed- and open-ended questions. The study was originally designed to compare weight and body composition of infants who were either exclusively breastfed or formula fed. It was later realized that infants in the formula feeding group received some amount of breastmilk and hence were classified as mixed feeding.

### 2.3. Anthropometric and Body Composition Measurements

Infant anthropometric measurements were conducted with a calibrated scale attached to the PEA POD for weight (Life Measurement Inc., Concord, Calif, USA) to the nearest 0.0001 kg and an infantometer (Seca 416) for length measurement to the nearest 0.1 cm. Both birthweight and birth length were reported by mothers.

Body composition made up of fat mass and fat-free mass was measured using the PEA POD body composition system following the manufacturers' protocol for body composition measurement (Life Measurement Inc. Concord, Calif, USA). The PEA POD is a pediatric air-displacement plethysmograph which assesses the body composition of infant birth through 6 months old by direct measurement of body mass and body volume using the principle of whole body densitometry. The description and operation of the PEA POD are reported elsewhere [20]. Before each body composition measurement, the infant's length is measured and inputted into the PEA POD computer system. The length imputed into the computer system is used to quantify the isothermal volume of air close to the infant's skin and lungs. All subjects were tested nude, and their hair was smoothed down with baby oil. This is to allow for precise quantification of the amount of air behaving isothermally in the test chamber to ensure accurate measurement according to the manufacturer of the PEA POD. Each body composition measurement in the test chamber lasted approximately 2 minutes. The within-day mean (±SD) reproducibility of infant percent body fat values measured with the PEA POD is 0.4 ± 1.8% in the Maternal and Child Nutrition Research Lab, Department of Foods and Nutrition, University of Georgia, Ga, USA.

### 2.4. Outcome Variables

The primary outcome variables were percent infant body fat and total body weight at the different time points. Changes in percent body fat and total body weight were estimated from one time point to the other.

### 2.5. Statistical Analysis

All data entry and analyses were performed using SPSS for Windows version 16.0 (SPSS Inc. Chicago, Ill, USA). Fat mass index (FMI) as a measure of infant adiposity was computed by the formula “fat mass in kilograms divided by the square of infant length in meters”, while fat-free mass index (FFMI) was computed by the formula “fat-free mass in kilograms divided by the square of infant length in meters”. Differences in variables by group were evaluated using *t*-tests and analysis of variance (ANOVA) for continuous variables. Chi-square analyses were used to examine bivariate associations between categorical variables. Repeated measures analysis was used to test the effects of type of infant feeding on weight and body composition (fat mass, fat-free mass, FMI, and FFMI) values. Pearson *r* was used to determine correlation between continuous variables and infant total body weight and percent body fat. The level for statistical significant was set at *P* = .05.

## 3. Results

### 3.1. Participant Characteristics

Of the 40 infants who participated in this study, 27 were exclusively breastfed, while the remaining 13 were mixed fed by 12 weeks postpartum. All infants in the mixed feeding group were introduced to infant formula on day 1 after delivery in addition to some breastmilk. The proportion of nonbreastmilk feed received by the mixed feeding group ranged from 10% to 100% of their total food intake across the study period. Majority of the infants in the mixed feeding group received on the average 50% to 70% of their total daily food intake from nonbreastmilk substitutes such as *Similac Advance, Isomil, Infamil, and Gerber Rice Cereal*. For the exclusively breastfeeding infants, they received only breastmilk as the sole source of nutrients, no water or other fluids through the 12 weeks of follow-up.

Descriptive statistics of the participants are presented in Table 1. Average age for mothers at delivery was 29.95 ± 5.04 (range 19–42) years with exclusively breastfeeding mothers being slightly older than their mixed feeding counterparts. Years of formal education were comparable between the two groups of mothers. Most of the mothers in both groups were married, white/Caucasian, and either employed full time or part time. Mean gestation age was 39.39 ± 0.87 (37–41) weeks, with no statistical difference in gestational age between the exclusively breastfeeding mothers and mixed feeding mothers and also by gender of the child (Table 1). There were no significant differences in both maternal and infant parameters between the two groups except for maternal prepregnancy weight, prepregnancy BMI, and maternal weight at delivery. Mothers of infants who received mixed feeding reported higher prepregnancy weight (*P* = .034), prepregnancy BMI (*P* = .011), and also weighed more at delivery (*P* = .033) than mothers of exclusively breastfeeding infants (Table 1). The infants were identical in birth weight and birth length in addition to gestational age. The mean birth weight was 3.47 ± 0.42 (range 2.51–4.26) kilograms, while mean birth length was 51.36 ± 2.06 (of range 46.99–54.61) centimeters. There were no significant differences in birth weight and birth length between exclusively breastfeeding and mixed feeding infants.

Vaginal/spontaneous delivery was higher among exclusively breastfeeding infants with cesarean delivery being higher among mixed feeding infants, although the differences were not statistically significant (Table 1).

### 3.2. Type of Feeding and Early Growth of Infant

**Figure 1:**
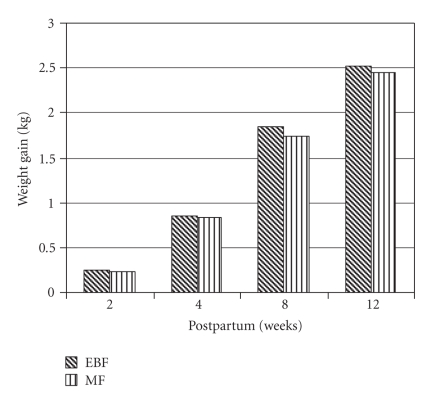
Changes in infant body weight with respect to birth weight (*EBF: exclusive breastfeeding; MF: mixed feeding*).

There was a modest increase in body weight across the feeding groups with time. Exclusively breastfeeding infants tended to gain slightly more weight with time compared to mixed feeding infants, although the differences in weight gain across time were not statistically significant (Figure 1). As depicted in Figure 1, the difference in weight gain between the two groups became more prominent at 8 (117.7 grams) and 12 (81.6 grams) weeks postpartum compared to the difference at 2 (16.9 grams) and 4 (12.7 grams) weeks postpartum with respect to birth weight. A similar trend was seen for infant length with exclusively breastfeeding infants gaining more length than mixed feeding infants even though the difference was not significant (data not shown).

### 3.3. Type of Feeding and Early Infant Body Composition

**Figure 2:**
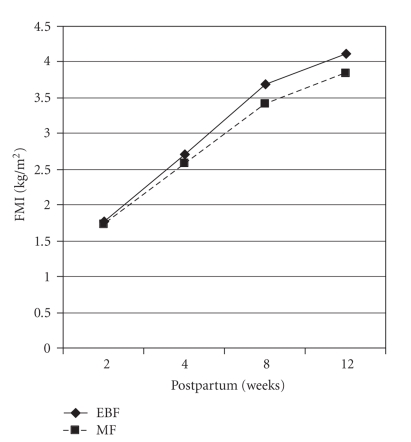
Pattern of infant adiposity with time (*EBF: exclusive breastfeeding; MF: mixed feeding*).

**Figure 3:**
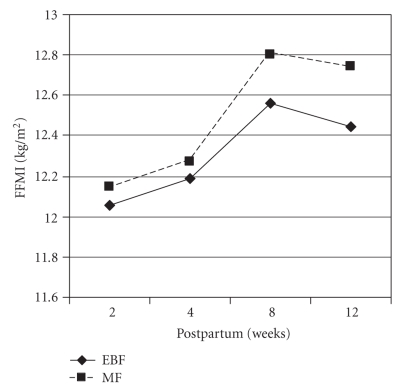
Pattern of infant lean mass with time (*EBF: exclusive breastfeeding; MF: mixed feeding*).

The results from this study showed gradual increase in the body composition (fat mass, fat-free mass, fat mass index [FMI], and fat-free mass index [FFMI]) of infants with time. There were, however, some differences in the accumulation of fat mass and fat-free mass. Exclusively breastfeeding infants accumulated more fat mass compared to their mixed feeding counterparts, whereas mixed feeding infants gained more fat-free mass compared to exclusively breastfeeding infants, although the difference was not statistically significant (data not shown). As shown in Figure 2, both groups started with about the same value for FMI at 2 weeks postpartum but with time the data showed slight differences although these were not statistically significant. Further examination of the data showed that exclusively breastfeeding infants gained slightly more FMI at 4 (0.93 kg/m^2^ versus 0.84 kg/m^2^), 8 (1.92 kg/m^2^ versus 1.68 kg/m^2^), and 12 (2.34 kg/m^2^ versus 2.10 kg/m^2^) weeks postpartum with respect to their FMI as computed at 2 weeks postpartum compared to mixed feeding infants (Figure 2). The results, therefore, show that by 12 weeks postpartum, infants who are exclusively breastfeeding have a mean FMI which is at least 0.2 unit higher than infants who are mixed feeding which is also an indication of higher adiposity among exclusively breastfeeding infants compared to mixed feeding infants. For FFMI which is a measure of infant lean mass, Figure 3 shows that unlike adiposity mixed feeding infants tended to have higher amounts of lean mass at 2 weeks postpartum and throughout the 12 weeks of follow-up compared to the exclusive breastfeeding counterparts. Mean FFMI gains were 0.13 kg/m^2^ versus 0.12 kg/m^2^, 0.51 kg/m^2^ versus 0.65 kg/m^2^, and 0.39 kg/m^2^ versus 0.60 kg/m^2^ at 4, 8, and 12 weeks postpartum with respect to FFMI at 2 weeks postpartum for exclusively breastfeeding and mixed feeding infants, respectively.

The repeated measures analysis did not show any significance between group differences in infant weight, length, fat mass, fat-free mass, FMI, FFMI, and percent body fat. Adjusted for birthweight, birth length and infant gender, fat mass, fat-free mass, FMI, and FFMI did not differ significantly between exclusively breastfeeding and mixed feeding infants at 2, 4, 8, and 12 weeks postpartum. There was a strong correlation between the computed FMI and the measured percent body fat (*r* = 0.949 to 0.974; *P* < .001) at each time point.

## 4. Discussion

The results from this pilot prospective study suggest that exclusive breastfeeding may promote increased weight gain and FMI in the early postpartum period (first 12 weeks after delivery) compared to the mixed feeding. Although the results of infant body composition measurement from this pilot studyare similar to previously reported data by Fomon et al. [21], Butte et al. [22], and Gilchrist [23], slight differences in infant feeding practices and infant growth and body composition are seen as they compared exclusive breastfeeding and formula feeding with the current study comparing exclusive breastfeeding to mix feeding. In agreement with the study by Gilchrist [23], no significant gender differences in weight gain and increase in adiposity were observed in this study, irrespective of the feeding type during the early postpartum weeks. This finding contradicts the findings by Buyken et al. [24] who studied older children. Buyken et al. found that male children exclusively breastfed for longer duration were protected against overweight/obesity even when their mothers were overweight [24]. In the study by Butte et al. [22], the authors observed higher weight velocity in formula fed than breastfed infants age 3 to 6 months, which is opposite to the findings from the current study of infants 0 to 12 weeks. Findings from the current study show that weight gain of exclusively breastfed infants was higher compared to their counterparts who received mixed feeding through the first 12 weeks after delivery (Figure 1), which is in agreement with previous studies, although they compared exclusive breastfeeding to formula feeding [25–27]. This means infants who are exclusively breastfed experience rapid weight gain during the first 12 weeks postpartum than infants who are either mixed or artificially fed. This is an interesting finding which needs further investigation beyond 12 weeks postpartum as we encourage more mothers to exclusively breastfeed their newborn for the first 6 months after delivery.

Also, the percent body fat in the exclusively breastfed infants exceeded that of mixed fed infants by 0.52% at 4 weeks postpartum, and the difference continued to increase throughout the study period, reaching 1.12% body fat at 12 weeks postpartum. The pattern of percent body fat gain observed in the present study is consistent with the findings by Gilchrist [23]. The same pattern was observed when FMI was used to assess infant adiposity (Figure 2). The difference in FMI and percent body fat between the feeding groups, although not statistically significant, is an important observation as it is a measure of adiposity. With the strong association of high adiposity with cardiovascular and other chronic diseases, it is important to understand why infants who are exclusively breastfed gain more percent body fat than those who do not receive exclusive breastfeeding in the first 12 weeks after delivery. It is also very important for future studies to document what happens to the differences in adiposity as infants double and triple their birthweight at 4–6 months and 9–12 months, respectively. In the present study, there was no direct measure of the infants' actual intake even though mothers who were mixed feeding reported proportion of daily feeds that came from nonbreastmilk substitute. It will be interesting and important for future studies to quantitatively estimate feed intake (breastmilk only versus breastmilk, and formula versus formula only) and their respective energy content to verify whether exclusively breastfeeding infants have higher intakes than the other feeding groups during early postpartum leading to the rapid weight and FMI/percent body fat gains. It will also be important to understand the metabolism of infants receiving different feedings during the early postpartum period. A better knowledge of the actual food intake and metabolic rate during the early postpartum period will help to explain why exclusively breastfeeding infants gain more body weight and FMI as well as percent body fat than infants receiving mixed or artificial feeding within the first 3 months of life. This information will also help in understanding why exclusive breastfeeding seems to promote rapid weight gain and high adiposity in the early postpartum period, while other studies find a protective effect or no effect on overweight/obesity later in the child's life.

Using the body mass index (BMI) as a proxy for assessing obesity and adiposity, a number of studies have reported the protective effect of breastfeeding on childhood obesity [28, 29]. These studies have been cross-sectional in nature and have relied on maternal/caretaker recall on infant feeding practices. Studies that have been prospective in nature in this area have either reported no effect of breastfeeding on overweight/obesity [30] or breastfeeding as a risk factor for overweight/obesity [31], while studies that employed meta-analysis have reported slight to moderate protection of breastfeeding against overweight/obesity during childhood [3, 5, 12, 17]. All these studies assessed the relationship between breastfeeding and overweight/obesity among older children and not the early postpartum period. Although, BMI tends to be a good screening tool for overweight/obesity, it is not enough, particularly when assessing adiposity hence the contribution of the current study using FMI in this area of research in the early postpartum period. By using FMI, fat mass was adjusted by infant length which is a fat-free proxy and not directly associated with body weight. Besides the weight and body composition measurements in this area of research, the definition of breastfeeding and duration may play important roles in the inconsistencies reported in the literature. Even though the current study has a small sample size, it is prospective in design. It applied the strictest definition for exclusive breastfeeding and also employed the PEA POD body composition system which has been proven to be accurate in the measurement of body composition in infants [20]. There is, therefore, a need for a prospective study with adequate power following children from early infancy through at least adolescence using well-defined breastfeeding definitions and dietary intake measures as well as using weight, BMI, and body composition methodologies that is proven to be accurate.

Findings from the current study alongside others suggest rapid weight gain and accumulation of adipose tissue in the early postpartum period which require further examination of the actual intake and metabolism of the infant to guide feeding and infant care recommendations given to mothers and caregivers as a means of preventing overweight and adiposity in children. It is important to emphasize that the study was designed to compare exclusive breastfeeding and formula feeding infants and weight and percent body fat accumulation, but the findings compare exclusive breastfeeding and mixed feeding instead. The results from this study need to be interpreted and generalized with caution because of the small sample and the participants being mainly whites and well educated. This study is one of the few that has examined the association between breastfeeding and childhood overweight/obesity by actually measuring infant body composition in the early postpartum period. With this said, practitioners and parents/caregivers should not lose sight of the numerous health benefits of breastfeeding to the infant, mother, and the entire society even during the early postpartum period. Also, the longer-term consequences of breastfeeding on body composition have a greater public health relevance than the effect within the first 12 weeks postpartum, requiring further prospective studies.

## 5. Conclusion

The rate of both weight gain and adiposity was slightly higher among infants who were exclusively breastfeeding compared to their counterparts who received mixed feeding in the first 12 weeks after delivery. Overall, exclusively breastfeeding infants had percent body fat which was 1.3% higher than mixed feeding infants at 12 weeks postpartum. Exclusively breastfeeding infants also weighed slightly more and had slightly higher FMI, less FFMI at 12 weeks postpartum compared to their mixed feeding counterparts. There is, therefore, a need for further prospective studies in this area to examine what happens to the percent body fat at 6 months and 12 months postpartum as infants are expected to double and triple their birth weight, respectively, as 6 months and 12 months postpartum are also high energy demand periods in the infant's life. This, if addressed, will contribute to the search for effective ways of overweight/obesity prevention especially in children. It will also give a better understanding of the differences in adiposity between exclusively breastfeeding, mixed feeding, and formula feeding infants in the early postpartum period.

## Figures and Tables

**Table 1:** Characteristics of mothers and infants according to type of feeding.

Maternal characteristics	EBF (*n* = 27)	MF (*n* = 13)	*P*-value
Age (years)	30.78 ± 5.16	28.23 ± 4.48	NS
Years of schooling	17.44 ± 2.26	17.31 ± 3.30	NS
Maternal height (m)	1.64 ± 0.07	1.61 ± 0.06	NS
Maternal prepregnancy weight (kg)	59.90 ± 8.28	67.27 ± 12.86	.034
Prepregnancy BMI (kg/m^2^)	22.21 ± 2.77	26.15 ± 6.59	.011
Maternal weight at delivery (kg)	75.13 ± 9.68	82.66 ± 10.91	.033
Pregnancy weight gain (kg)	15.10 ± 3.95	15.42 ± 4.52	NS
Gestational age at delivery (weeks)	39.46 ± 0.86	39.25 ± 0.90	NS

	*n* (%)	*n* (%)	

Marital Status			NS
Married	25 (92.6)	11 (84.6)	
Single	2 (7.4)	2 (15.4)	

Ethnicity			NS
White/Caucasian	24 (88.9)	10 (76.9)	
Black/African American	3 (11.1)	3 (23.1)	

Employment status			NS
Full time	15 (55.6)	8 (61.5)	
Part time	7 (25.9)	4 (30.8)	
Not working	5 (18.5)	1 (7.7)	

Type of delivery			NS
Vaginal/spontaneous	23 (85.2)	8 (69.2)	
Cesarean	4 (14.8)	4 (30.8)	

Infant characteristics			
Birth weight (kg)	3.44 ± 0.42	3.52 ± 4.33	NS
Birth length (cm)	51.36 ± 2.03	51.34 ± 2.20	NS

Gender			NS
Boys	15 (55.6)	7 (53.8)	
Girls	12 (44.4)	6 (46.2)	

*EBF: exclusive breasfeeding; MF: mixed feeding*.
